# Randomized comparison of the effects of tailored text messaging versus pillbox organizers on medication adherence of heart failure patients

**DOI:** 10.1186/s12872-024-03884-1

**Published:** 2024-05-09

**Authors:** Ameneh FarzanehRad, Atefeh Allahbakhshian, Leila Gholizadeh, Azizeh Farshbaf Khalili, Hadi hasankhani

**Affiliations:** 1 Faculty of Nursing and Midwifery, Tabriz University of Medical Sciences Tabriz, Tabriz, East Azerbaijan Iran; 2 Faculty of Health, University of Technology Sydney, PO Box 123, Broadway, Sydney, NSW 2007 Australia; 3 Physical Medicine and Rehabilitation Research Centre, Tabriz University of Medical Sciences, Tabriz, East Azerbaijan Iran; 4 School of Nursing & Midwifery, Tabriz University of Medical Sciences, Tabriz, Iran

**Keywords:** Medication adherence, Text message reminders, Text messaging, Pillbox organizers, Heart failure

## Abstract

**Background:**

Heart failure (HF) is a major public health issue worldwide, affecting approximately 64.3 million people in 2017. Non-adherence to medication is a common and serious issue in the management of HF. However, new reminder systems utilizing mobile technology, such as text messaging, have shown promise in improving medication adherence. The purpose of this study was to compare the impact of tailored text messaging (TTM) and pillbox organizers on medication adherence in individuals with HF.

**Methods:**

A randomized controlled trial was conducted, involving 189 eligible patients with HF who were randomly assigned to either the TTM, pillbox organizer, or control group. Medication adherence was evaluated using pill counting and the Medication Adherence Rating Scale (MARS) over a period of three months and compared across the groups. The data were analyzed using Kruskal-Wallis, Analysis of Variance (ANOVA), and Repeated Measures ANOVA tests.

**Results:**

The results indicate that both the TTM and pillbox organizers groups had significantly higher medication adherence compared to the control group, as measured by pill counting (MD = 0.05, 95%CI = 0.03–0.06; *p* < 0.001 for TTM group, MD = 0.04, 95%CI = 0.03–0.06; *p* < 0.001 for pillbox organizers group) and the MARS (MD = 1.32, 95%CI = 0.93 to 1.72; *p* < 0.001 for TTM group, MD = 1.33, 95%CI = 0.95 to 1.72; *p* < 0.001 for pillbox organizers group). However, there was no statistically significant difference in medication adherence between the two intervention groups using either measurement method. The TTM group exhibited a lower hospitalization rate than the other groups in the first follow up (*p* = 0.016).

**Conclusions:**

Both the TTM and pillbox organizers were shown to be effective in enhancing medication adherence among patients with HF. Therefore, healthcare providers should take into account the patient’s condition and preferences when selecting one of these methods to promote medication adherence. Future research should aim to address the limitations of this study, such as controlling for confounding variables, considering long-term effects, and comparing the effectiveness of different interventions.

## Background

Heart failure (HF) is a major public health issue worldwide, affecting approximately 64.3 million people in 2017, with the highest rates among middle east countries [1]. This disease is linked to a higher risk of mortality, decreased quality of life, and substantial economic burden on individuals and societies. Despite the recent progress in treating HF, which has led to better patient survival rates, the overall prognosis for this condition is still unfavourable [2]. The one-year mortality rate following hospitalization for HF is reported to be 25-30%, surpassing the rates associated with numerous common cancers ( [2]. However, research indicates that adhering to treatment regimens, including medication adherence, can significantly improve the prognosis of HF [3]. Patients who comply with their prescribed medication have lower rates of disease-related complications and hospitalizations [4]. Nonetheless, non-adherence rate among this patient group ranges from 5 to 60%, with higher non-adherence rates observed in low-income countries [5].

In Iran, CVD is responsible for approximately 46% of all deaths; however, reliable estimates of the incidence and prevalence of HF in this region are currently unavailable [6]. Additionally, lower adherence to HF medications is a concern in this country, as more than half of the patients demonstrate low medication adherence [7].

Failing to follow prescribed HF medication regimens is linked to the exacerbation of HF symptoms, reduced physical functioning, hospital readmissions, mortality, and higher healthcare costs [3]. Hospital readmissions are frequent in HF patients, with around 25% of them being readmitted within 30 days of discharge and approximately 50% within six months [8]. Medication non-adherence is responsible for an estimated 10% of readmissions [9]. Non-adherence to HF medication is multifactorial, including socioeconomic, psychological, cognitive, disease and therapy, and health care factors [10–12].

Various strategies have been studied to overcome barriers to non-adherence to HF medications. One potentially cost-effective approach is the use of pillbox organizers [13]. Pillboxes are containers that can help patients organize and store scheduled doses of medications. Simple pillboxes are readily available and can make the task of taking medications easier for patients [14]. Previous research has shown the positive effects of using pillboxes on medication adherence [13, 15].

Another approach that has been explored is text messaging through mobile phones, which can be particularly useful in low-income countries where mobile phones are a relatively inexpensive method of communication [16]. Text message-based interventions offer a simple, cost-effective, acceptable, and feasible approach to improving medication adherence, whether by using medication reminders or supportive messages [17]. Mobile applications are favoured over traditional mobile phone messaging for sending text messages due to their capacity for pre-sending, scheduling, enhanced automation, and monitoring capabilities [18, 19], . Through mobile applications, relevant information can be sent to the text message reminder system via the internet in a personalized manner according to the patient’s medication regimen at specific time intervals.

A meta-analysis has shown that text messaging interventions can effectively improve medication adherence, with patients having nearly double the odds of achieving medication adherence [20, 21].

While this method has proven successful in enhancing medication adherence across various health conditions, its effectiveness in HF patients has not been sufficiently examined, and the results from previous studies have been inconsistent [22, 23]. The notable characteristic of this research is its methodology, which incorporated the expertise of an information technology consultant to dispatch automated and personalized text message reminders to patients exactly at their designated medication intake times. Previous studies on HF patients either lacked personalization or did not specifically focus on medication adherence. Additionally, they were mostly conducted in developed and resource-rich countries. In addition, the pill box organizers were prepared by the researchers and not patients in our study. These methods may better suit patients with HF due to cognitive impairment associated with HF [24]. This study aimed to compare the effect of tailored text messaging to pillbox organizers on medication adherence in HF patients.

## Methods

### Study design and participant recruitment

This single-blind randomized clinical trial aimed to investigate and compare the effect of tailored text messaging and pillbox organizer on medication adherence in patients with HF. The study recruited eligible participants from a tertiary referral cardiovascular center in Iran between 1.08.2021 and 21.09.2021. Subsequently, the participants underwent a follow-up period lasting for 3 months.

### Inclusion and exclusion criteria

Participants were selected based on the following inclusion criteria: being at least 18 years of age, having a diagnosis of HF classified as II or III according to the NYHA Functional Classification [25], demonstrating the ability to manage medications independently, having proficiency in reading and writing in Farsi, having access to a mobile phone, and providing informed consent to take part in the study. Participants with a score of less than 7 on the AMTS were excluded from the study.

### Sample size calculation

The required sample size for the current study was calculated based on the results of a similar study conducted previously [18]. Using the formula for comparing two proportions, with P1 = 0.68 and P2 = 0.88, a 95% confidence level, 80% power, and a two-tailed test, it was determined that a sample size of 57 participants per study arm was needed. Accounting for a potential attrition rate of 10%, the study recruited a total of 189 participants, with 63 participants assigned to each study arm.

### Randomization

This study was registered with the Iranian Registry of Clinical Trials and approved by a regional ethics committee affiliated with Tabriz University of Medical Sciences (IR.TBZMED.REC.1399.837). The researcher provided eligible participants with information regarding the study and its objectives. Assurance was given regarding the confidentiality of their information and the safety of the study interventions. Participants were then randomly assigned to one of three groups: tailored text messaging (TTM), pillbox organizer (PO), or control, in a 1:1:1 ratio. Random numbers were generated using a random number generator software. Allocation concealment was achieved by using identical sealed opaque envelopes numbered sequentially from 1 to 189. The production of allocation sequence and preparation of envelopes were carried out by an independent person. The statistician who analyzed the data and outcome assessors were blind to the group allocations. Due to the nature of the study, it was not possible to blind the participants and the researchers.

### Tailored text messaging (TTM)

In the initial meeting with participants in the (TTM) group, they filled out the MARS questionnaire. Subsequently, the researcher conducted training on the usage of the text messaging system and sought feedback to ensure that participants could readily read and comprehend an example text message. The text messages were sent using a mobile application that automatically send medication reminders several times a day based on each individual participant’s medication regimen. The messages were designed to be easily understood and limited in the number of words. Participants could receive and read the messages on any mobile phone used in the country’s mobile network. An example text message was “Hi Mr. Ahmadi, Please take 10 mg of Lasix at 8 am”.

At the start of the trial, the researchers provided participants with a one-month supply of medication in a simple box, personally handing it to them. Participants were instructed to visit the center at the end of each month. During these visits, researchers recorded the remaining pills, dispensed medications for the next month, and asked participants to complete the MARS.

### Pillbox organizer

Participants in this group completed the MARS questionnaire and subsequently were provided with four pill box organizers, with each box corresponding to a week of medication. The researchers placed the participant’s medication in the pillbox organizers, which had four compartments for different times of the day: morning, noon, evening, and night. Participants received training on how to use the pillbox organizers, and their ability to use the organizers was assessed. The researchers also provided their telephone number to participants in case they needed assistance. Participants were asked to return to the center at the end of each month, for three consecutive months, to have their remaining pills counted, have their pillboxes refilled for the next four weeks, and complete the MARS questionnaire.

### Control group

As a routine care, each participant in control group and other groups received personalized, face-to-face instruction from a specialist nurse regarding heart failure, emphasizing the necessity for lifestyle modifications. Comprehensive education was provided on their prescribed medications, covering details such as drug types, purposes, dosage, frequency, potential drug interactions, and associated side effects.

The participants in the control group then completed the MARS questionnaire and received their medications for a month in a simple box provided by the researcher. The simple box served as a container for storing monthly medicines and had no additional function. Like the other groups, these participants were asked to return to the center at the end of each month for three consecutive months. The researchers counted their remaining pills, provided them with medications for the next month, and asked them to complete the MARS questionnaire.

### Measurements

The data collection tools used in this study included several forms: a sociodemographic and clinical characteristics form, the MARS, the NYHA Functional Classification, and the AMTS. The sociodemographic and clinical characteristics form was developed by the researchers to gather information on participants’ demographics, including age and gender, social characteristics such as job and education and clinical information, such as history of HF and ejection fraction. At the beginning of each month, the researchers recorded the name and number of medications taken by each participant, as well as whether they were admitted to the hospital for HF during the preceding month. Ejection fractions and HF classes, determined according to the NYHA Functional Classification, were extracted from participants’ medical records.

To assess medication adherence status, the researchers counted pills and used the MARS. The MARS is a 10-item self-report tool designed to assess patients’ adherence to their medications. Participants responded with either ‘yes’ or ‘no’ to each item, with a ‘yes’ response scored zero for items 1–6 and 9–10, and a ‘no’ response scored one. The scoring is reversed for items 7 and 8.

The total medication adherence score for each participant is calculated by adding up the scores to individual items, with higher scores indicating greater adherence. A total score of 7–10 indicates high adherence, 4–6 indicates moderate adherence, and 0–3 indicates low adherence. The validity of the MARS has been confirmed on a wide range of diseases and patient groups [26, 27]. In this study, the internal consistency of the scale was determined to be high, with a Cronbach’s alpha coefficient of 0.84. To assess medication adherence, the researchers counted the number of remaining pills at the end of each month for three consecutive months, and participants completed the MARS at baseline, as well as at the end of the first, second, and third month.

Participants’ cognitive status was evaluated using the AMTS. This tool is widely used to assess a cognition on hospital admission. Which is a 10-item screening tool originally designed to identify possible dementia in elderly patients. A score of one is given for each correct answer, resulting in a total score ranging from 0 to 10. A total score of 0–3 indicates severe cognitive impairment, 4–6 indicates moderate cognitive impairment, and scores higher than 7 indicate normal cognitive status [28]. The validity of this tool has been established for assessing cognitive impairment in patients with various health conditions and ethnic backgrounds [29–31]. The Persian version of the AMTS has been validated on an elderly Iranian sample, indicating that the test is suitable for use with Persian-speaking older adults in Iran [32].

### Study outcomes

The primary outcome of the study was to evaluate the improvement in medication adherence at the three-month follow-up, which was measured by counting pills. The secondary outcomes included improvements in medication adherence, as assessed by counting pills and the MARS, at different follow-up time points, as well as a reduction in hospitalization rates.

### Data analysis

The Statistical Package for the Social Sciences (SPSS) version 24 was used to analyze the data. The normality of quantitative data was tested and confirmed using the Kolmogorov-Smirnov test. Descriptive statistics such as frequency, percentage, and measures of central tendency were used to summarize the data. The baseline similarity of the groups in terms of sociodemographic and clinical characteristics was compared using t-test, one-way ANOVA, and chi-square test. Adherence to medications was compared between the groups using one-way ANOVA with Tuckey post hoc test. The changes in medication adherence over time were assessed and compared between the groups using repeated measures ANOVA with Fisher’s least significant difference (LSD) post hoc test, while controlling for age and income status. The proportions of participants who adhered to medications were compared across the groups using Kruskal-Wallis and Mann-Whitney tests. Chi-square test was used to test for differences in hospitalization rates between the groups. The significance level was set at *p* < 0.05.

## Results

**Fig. 1:**
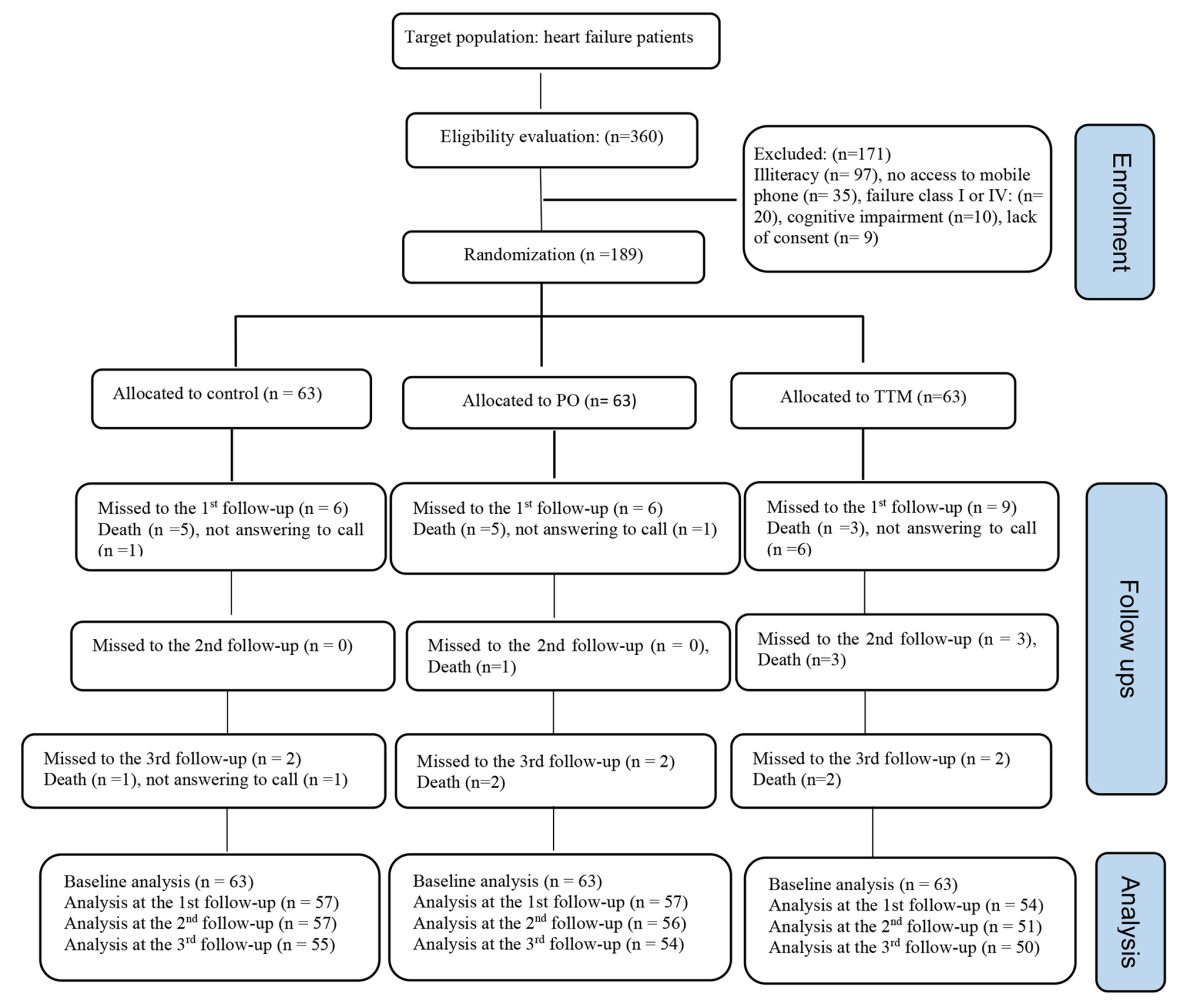
The participants’ flow diagram

A total of 360 patients were screened for inclusion in the study. Of these, 171 patients did not meet the inclusion criteria due to various reasons, including inability to read and write (*n* = 97), lack of access to a mobile phone (*n* = 35), not having a diagnosis of HF class II or III (*n* = 20), cognitive impairment (*n* = 10), and unwillingness to participate in the study (*n* = 9). The participants’ flow diagram is illustrated in Fig. 1. The mean ± standard deviations (SD) of age of participants in tailored text messaging, pillbox organizer, and control groups were 61.7 ± 13.0, 64.2 ± 8.9, and 59.8 ± 10.5 years, respectively. Participants were mostly male in all groups; TTM (68.3%), pillbox organizer (65.1%), and control group (71.4%). The duration of HF disease illness was less than one year in nearly half of participants; TTM (49.2%), pillbox reminder (42.9%), and control (44.4%). Participants’ sociodemographic and clinical characteristics are demonstrated in Table 1. The study groups were homogeneous in sociodemographic and clinical characteristics, except for age (*p* = 0.028), and economic status (*p* = 0.001).

**Table 1:** Comparison of the sociodemographic and disease characteristics in the study groups

Variables	TTM (*n* = 63)	PO (*n* = 63)	Control (*n* = 63)	Total (*n* = 189)	Statistical indices
**Age (Year**)/n (%)					
< 45 years ≥ 46 years	(11.1%) 56 (88.9%)	0 (0.0%) 63 (100.0%)	4 (6.3%) 59 (93.7%)	11 (5.8%) 178 (94.2%)	*χ*^*2*^ = 7.14, df = 2 ***p*** **= 0.028**
**Sex** /n (%)					
female male	20 (31.7%) 43 (68.3%)	22 (34.9%) 41 (65.1%)	18 (28.6%) 45 (71.4%)	60 (31.7%) 129 (68.3%)	*χ*^*2*^ = 0.58, df = 2 *p* = 0.746
**Marital status**/n (%)					
Single and widowed Married	8 (12.7%) 55 (87.3%)	4 (6.3%) 59 (93.7%)	5 (7.9%) 58 (92.1%)	17 (9.0%) 172 (91.0%)	*χ*^*2*^ = 1.68, df = 2 *p* = 0.432
**Education/***n* (%)					
Reading and writing High school ≥Diploma	18 (28.6%) 29 (46.0%) 16 (25.4%)	25 (39.7%) 24 (38.1%) 14 (22.2%)	15 (23.8%) 35 (55.6%) 13 (20.6%)	58 (30.7%) 88 (46.6%) 43 (22.8%)	Linear-by-Linear = 0.001 df = 1, *p* > 0.999
**Job/***n* (%)					
Others Employee Housewife Unemployed Retired	24 (38.1%) 5 (7.9%) 20 (31.7%) 4 (6.3%) 10 (15.9%)	22 (34.9%) 4 (6.3%) 20 (31.7%) 2 (3.2%) 15 (23.8%)	28 (44.4%) 3 (4.8%) 15 (23.8%) 4 (6.3%) 13 (20.6%)	74 (39.2%) 12 (6.3%) 55 (29.1%) 10 (5.3%) 38 (20.1%)	χ2 = 3.96, df = 8 *p* = 0.860
**Income/n (%)**					
Income < expenses Income ≈ expenses Income > expenses	6 (9.5%) 56 (88.9%) 1(1.6%)	11 (17.5%) 51 (81.0%) 1(1.6%)	24 (38.1%) 38 (60.3%) 1(1.6%)	41 (21.7%) 145 (76.7%) 3 (1.6%)	Linear-by-Linear = 13.29 df = 1, ***p*** **= 0.001**
**Duration of illness/***n* (%)					
> 1 years 1–5 years 6–10 years >10 years	31 (49.2%) 13 (20.6%) 11 (17.5%) 8 (12.7%)	27 (42.9%) 14 (22.2%) 9 (14.3%) 13 (20.6%)	28 (44.4%) 17 (27.0%) 12 (19.0%) 6 (9.5%)	86 (45.5%) 44 (23.3%) 32 (16.9%) 27 (14.3%)	Linear-by-Linear = 0.001 df = 1, *p* > 0.999
**Ejection fraction** % (Mean ± SD)	29.04 ± 8.97	26.82 ± 9.76	26.74 ± 7.52	27.53 ± 8.82	ANOVA df = 2, F = 1.38, *p* = 0.252
**History of hospitalization** /n (%)					
Yes No	47 (74.6%) 16 (25.4%)	48 (76.2%) 15 (23.8%)	47 (74.6%) 16 (25.4%)	142 (75.1%) 47 (24.9%)	χ2 = 0.05, df = 2, *p* = 0.972

PO: Pillbox organizers; TTM tailored text messaging

### Medication adherence

Statistically significant differences in medication adherence, as measured by pill counting, were found between the groups. A significantly higher proportion of participants in both the TTM group (*p* < 0.001) and the Pillbox organizers group (*p* < 0.001) adhered to their medication compared to those in the control group. However, there was no significant difference in medication adherence between the TTM and Pillbox organizers groups based on pill counting (*p* = 0.978) (Table 2).

**Table 2:** Comparison of medication adherence based on pill counting (*n* = 63 participnts in each group)

Medication adherence	TTM Mean ± SD	PO Mean ± SD	Control Mean ± SD	*p* value^a^	*p* value^b^	*p* value^c^
**1st fu**	0.98 ± 0.02	0.96 ± 0.02	0.91 ± 0.08	**< 0.001**	**< 0.001**	**0.134** ^e^
**2nd fu**	0.97 ± 0.02	0.97 ± 0.01	0.92 ± 0.06	**< 0.001**	**< 0.001**	0.929
**3rd fu**	0.96 ± 0.08	0.97 ± 0.02	0.93 ± 0.04	**< 0.001** ^e^	**< 0.016** ^e^	0.243
Total	0.97 ± 0.04	0.97 ± 0.01	0.92 ± 0.06	**< 0.001** ^d^	**< 0.001** ^d^	0.978^d^

TTM: Tailored text messaging group; Po: pillbox organizers; *p* value^a^: comparing mean differences between PO and control; *p* value^b^: comparing mean differences between TTM and control; *p* value^c^: comparing mean differences between PO and TTM.

**Fig. 2:**
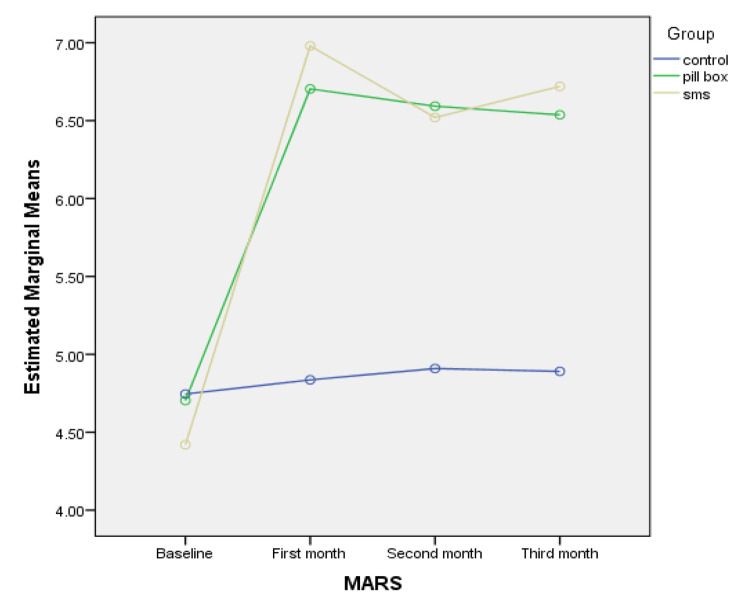
Changes in medication adherence based on the MARS results over three months period

The MARS scores did not differ significantly between the study groups (*p* > 0.05). After interventions, there was a statistically significant interaction effect between time and group (*p* < 0.05), indicating that participants in the TTM (MD = 1.32, 95%CI = 0.93 to 1.72; *p* < 0.001) and pillbox organizers groups (MD = 1.33, 95%CI = 0.95 to 1.72; *p* < 0.001) reported higher medication adherence than those in the control group. The difference in the MARS scores between participants in the TTM and pillbox organizers groups was not also statistically significant (MD=-0.01, 95%CI= -0.39 to 0.37, *p* = 0.952) (Table 2; Fig. 2).

The proportion of participants who were non-adherent, partially adherent, and adherent in each group based on the MARS results is presented in Table 3. Consistent with the previous results, there was no significant difference in medication adherence between the study groups at baseline (*p* = 0.255). The results showed significant differences in medication adherence between the groups at the end of the first (*p* < 0.001), second (*p* < 0.001), and third months (*p* < 0.001). A higher proportion of participants in the TTM and pillbox organizers groups were adherent to their medications compared to the control group. However, there was no statistically significant difference in medication adherence between the TTM and pillbox organizers groups at any of the follow-up time points. Notably, none of the participants in either intervention group were classified as “non-adherent” in any of the follow-up points (Table 3).

**Table 3:** Comparison of hospitalization rates among study groups

Hospitalization	TTM (*n* = 63)	Pill box (*n* = 63)	Control (*n* = 63)	Total (*n* = 189)	Statistical indices
Baseline/*n* (%)					
yes no	47(74.6%) 16(25.4%)	48(76.2%) 15(23.8%)	47(74.6%) 16(25.4%)	142(75.1%) 47(24.9%)	χ2 = 0.05, df = 2, *p* = 0.972
The 1-month follow up /*n* (%)					
yes no	7(13.0%) 47(87.0%)	19(33.3%) 38(66.7%)	9(15.8%) 48(84.2%)	35(20.8%) 133(79.2%)	χ2 = 8.30, df = 2, ***p*** **= 0.016**
The 2-month follow up /*n* (%)					
yes no	5(9.8%) 46(90.2%)	7(12.5%) 49(87.5%)	1(1.8%) 56(98.2%)	13(7.9%) 151(92.1%)	χ2 = 4.82, df = 2, *p* = 0.090
The 3-month follow up /*n* (%)					
yes no	4(8.0%) 46(92.0%)	3(5.6%) 51(94.4%)	5(9.1%) 50(90.9%)	12(7.5%) 147(92.5%)	χ2 = 0.51, df = 2, *p* = 0.775

TTM: Tailored text messaging group

Finally, the hospitalization rates among the groups showed a statistically significant difference (*p* = 0.016) only in the first follow-up. In this follow-up, the TTM group exhibited a lower hospitalization rate than the other groups, as shown in Table 4.

**Table 4:** Comparison of medication adherence based on counting pills during study

Medication adherence (0–10)	SMS (*n* = 63) Mean ± SD	Pill box (*n* = 63) Mean ± SD	Control (*n* = 63) Mean ± SD	^a^ Mean difference (95% CI) *P*-value	^b^ Mean difference (95% CI) *P*-value	^c^ Mean difference (95% CI) *P*-value
First month	0.98 ± 0.02	0.96 ± 0.02	0.91 ± 0.08	0.05 (0.03 to 0.07) **< 0.001** ^€^	0.07 (0.05 to 0.09) **< 0.001** ^€^	0.01 (-0.005 to 0.03) **0.134** ^€^
Second month	0.97 ± 0.02	0.97 ± 0.01	0.92 ± 0.06	0.05 (0.03 to 0.07) **< 0.001** ^€^	0.05 (0.03 to 0.07) **< 0.001** ^€^	0.001 (-0.01 to 0.01) 0.929 ^€^
Third month	0.96 ± 0.08	0.97 ± 0.02	0.93 ± 0.04	0.04 (0.01 to 0.06) **< 0.001** ^€^	0.02 (0.005 to 0.051) **< 0.016** ^€^	0.01 (-0.03 to 0.009) 0.243 ^€^
Total	0.97 ± 0.04	0.97 ± 0.01	0.92 ± 0.06	0.04 (0.03 to 0.06) **< 0.001**¥	0.05 (0.03 to 0.06) **< 0.001**¥	0.001 (-0.01 to 0.01) 0.978¥

^a^: The difference between the pillbox and the control groups b: The difference between the SMS and the control groups C: The difference between the SMS and the pillbox group

¥ ANOVA test with repeated measurement using LSD post hoc test adjusted for baseline values, age, income, time-group interaction was significant (Greenhouse-Geisser: p = 0.032)

€ Analysis of covariance adjusted for baseline values, age, income

## Discussion

The objective of this study was to compare the effectiveness of TTM and pillbox organizers on medication adherence and hospitalization rates in HF patients. The results of the study showed that both TTM and pillbox organizers were effective in improving medication adherence when compared to participants who received their medications monthly in a simple box. This was confirmed by both the (MARS) scores and pill counting, which is considered a more accurate method of assessing medication adherence [33]. Additionally, none of the participants in the TTM and pillbox organizers group were classified as ‘non-adherent’ during any of the follow-up points.

These findings are consistent with previous research on the effectiveness of text message reminders on medication adherence in patients with various conditions, such as coronary heart disease [34], type II diabetes [35], bipolar I disorder [36], liver transplant [37], and asthma [38]. The results also support most previous studies that have reported the usefulness of pillbox organizers on medication adherence [9, 13, 15]. These findings suggest that text message reminders and pillbox organizers may be used as strategies to improve medication adherence in patients with chronic diseases, regardless of their diagnosis. Pillbox organizers can help reduce the chances of unintentional medication non-adherence caused by confusion about which medications to take on a given day or at a particular time. Pillboxes allow for the storage of medications in separate compartments for different days of the week and different times of the day [9, 13].

However, compiling evidence on the effectiveness of pillboxes on medication adherence can be challenging, as there are various types of pillbox devices available, with different levels of complexity for the patient. For instance, some smart pill organizers include alarm reminders. Some studies fail to provide a detailed explanation of their pillbox intervention (8). For example, in a study by Mehdinia et al. (2020), the effectiveness of pillbox organizers on medication adherence in elderly patients with cardiovascular disease was investigated. The participants were trained to fill their pillboxes at the end of each week for the following week. The pillboxes used had seven columns for a whole week labelled with weekday names and four cells for each day marked as morning, noon, and evening. In contrast, in the current study, the researchers prepared the pillboxes for participants each month (four pillboxes for four weeks) for three consecutive months.

The findings of this study are consistent with previous research reporting positive effects of text messaging on medication adherence in patients with chronic diseases [39]. However, this study is unique in that the text messages were tailored to each participant’s name and included information about the specific medications they were prescribed, including their dosage and when they should be taken.

Generic text messages can be perceived as intrusive, repetitive, and untimely, which may reduce their effectiveness as an intervention. Tailored text messages that are scheduled in consultation with the patient and consider their preferences may be more effective. Furthermore, these messages may be limited to instances when the patient has forgotten to take their medication, as detected by an electronic device [35].

Based on the findings of our study, healthcare providers may opt for either TTM or pillbox organizers to enhance adherence to medication regimens among HR patients. Since both methods proved effective in improving adherence to heart failure medications, factors such as patient preferences, abilities, and cost should be taken into consideration when selecting a support method. Pillbox organizers may be a more cost-effective option compared to TTM and may be suitable for patients without a mobile phone or reading ability. In our study, researchers filled the pillbox organizers at the beginning of each month. Healthcare providers, especially pharmacists, may consider implementing this approach for patients with cognitive impairments. TTM, while potentially more expensive, might be considered for individuals experiencing medication nonadherences due to forgetfulness.

In comparison to other studies reporting medication adherence rates in patients with HF in Iran, our study observed lower non-adherence rates. This discrepancy could be attributed, at least in part, to the coincidence of our study with the COVID-19 pandemic. The warnings from healthcare providers regarding the management and control of chronic diseases during the pandemic may have contributed to an increase in medication adherence among our study participants. Studies investigating the impact of COVID-19 on medication adherence in patients with chronic diseases have yielded inconsistent results. While some studies have reported a reduction in medication adherence due to factors such as contagion fears, failure to return to the hospital for prescription refills, missed doses, or dose reductions due to psychological stress, other studies have found a positive impact of the pandemic on medication adherence. This positive impact may be attributed to the increased availability of health information and advice during the pandemic [40].

Additionally, our study excluded illiterate patients, who are more likely to have poor medication adherence. This may have contributed to the higher medication adherence rates among participants. However, excluding illiterate patients may limit the generalizability of the study results, as not all patients with HF are literate.

### Study strengths and limitations

This study possesses significant strengths, foremost among them being its randomized controlled design and the meticulous incorporation of allocation concealment, thereby substantially reducing the potential for bias. Both the MARS and pill counting were employed to assess medication adherence, a dual strategy aimed at mitigating the risk of bias with self-reports [41]. While double blinding was impractical given the nature of the intervention, data analysis was carried out by a statistician who remained blind to the group allocations, enhancing the study’s objectivity.

Despite the impracticality of double-blinding due to the nature of the intervention, the data analysis was conducted by a statistician who remained blind to the group allocations, enhancing the integrity of the study. The randomized controlled design of this study and using allocation concealment are the major strengths of this study, reducing the risk of bias. We used both the MARS and pill counting to assess medication adherence, a strategy that reduced the risk of reporting bias. Furthermore, although double blinding was not possible due to the nature of the intervention, the data analysis was completed by a statistician who was blind to the group allocations. Nevertheless, it is important to acknowledge the potential for attrition bias, especially considering that 20.64% of participants from the TTM group were lost to follow-up. Additionally, we did not collect and control for some confounding variables, such as anxiety, depression, or overall physical health, which may affect medication adherence [42]. In this study, the method used to send tailored text messages was non-interactive and one-directional. We chose not to ask participants to confirm whether they had taken their medication after receiving the text message, as a precaution against potential social desirability bias. However, in future studies, it may be beneficial to evaluate this bias in participants’ interactions with text messaging by cross-referencing their self-reports with objective measures such as pill counting. A recent systematic review suggested that interactive eHealth interventions are effective in enhancing medication adherence in adults with long-term medication regimens. However, the review did not provide conclusive evidence on whether interactive eHealth interventions are more effective than non-interactive and one-directional interventions [43]. Therefore, it is recommended that future research compare the effect sizes of different text message interventions to identify an intervention which is more effective on medication adherence, cost-effective, and a preferred method by patients [43]. In our study, the researchers filled the pillbox organizers, and this practice may have contributed to the non-significant difference observed between the pillbox organizer and TTM groups. Subsequent studies might reveal a statistically significant difference in medication adherence between pillbox organizers and TTM if participants are tasked with filling their own pillbox organizers.

Additionally, we followed participants for only three months, and uncertainty remains about the long-term effects of such interventions, as patients may lose interest in text messages about their medications over the long term. Future research should address the long-term effectiveness of these interventions. Researchers may also explore the underlying reasons for the immediate reduction in hospitalization rates observed in the TTM group, as well as investigate whether this effect is sustained over the long term.

## Conclusion

This study adds to the growing body of literature that suggests the potential benefits of using technology-based interventions to improve medication adherence in patients with chronic diseases. Our study underscores the potential effectiveness of tailored text messages and pillbox organizers, prepared by healthcare providers, in enhancing medication adherence among patients with HF. Failure to adhere to prescribed medications is associated with heightened mortality, morbidity, increased rates of hospitalization, and amplified healthcare expenditures [44]. Future research endeavors should prioritize addressing the limitations identified in this study, with particular attention to controlling for confounding variables, especially in cases where pillbox organizers are prepared either by patients or healthcare providers. Furthermore, investigations should concentrate on evaluating the sustained impact of these interventions over the long term. The practical implementation of these interventions in real-world clinical settings necessitates careful consideration of factors such as patient preferences and cost-effectiveness to ensure widespread adoption and effectiveness.

## Data Availability

Please contact Associate professor Atefeh AllahBakhhsian for the data related to this study.
